# Supplementary material for: Influence of Antisynthetase Antibodies Specificities on Antisynthetase Syndrome Clinical Spectrum Time Course
Source: J Clin Med. 2019 Nov 18;8(11):2013. doi: 10.3390/jcm8112013 (PMC6912490; doi:10.3390/jcm8112013)
Supplement: Supplementary file 1 [file jcm-08-02013-s001.pdf]

Supplementary Materials

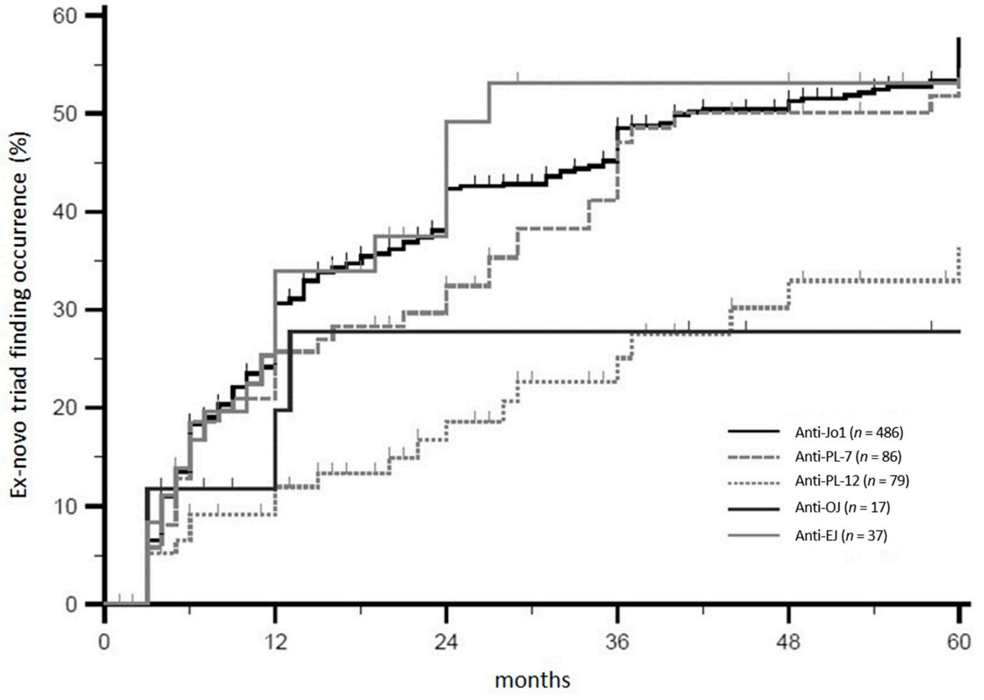


**Figure S1**: prevalence of ex-novo triad findings occurrence in our cohort of antisynthetase syndrome patients according to follow-up lenght (established range: 0–60 months)

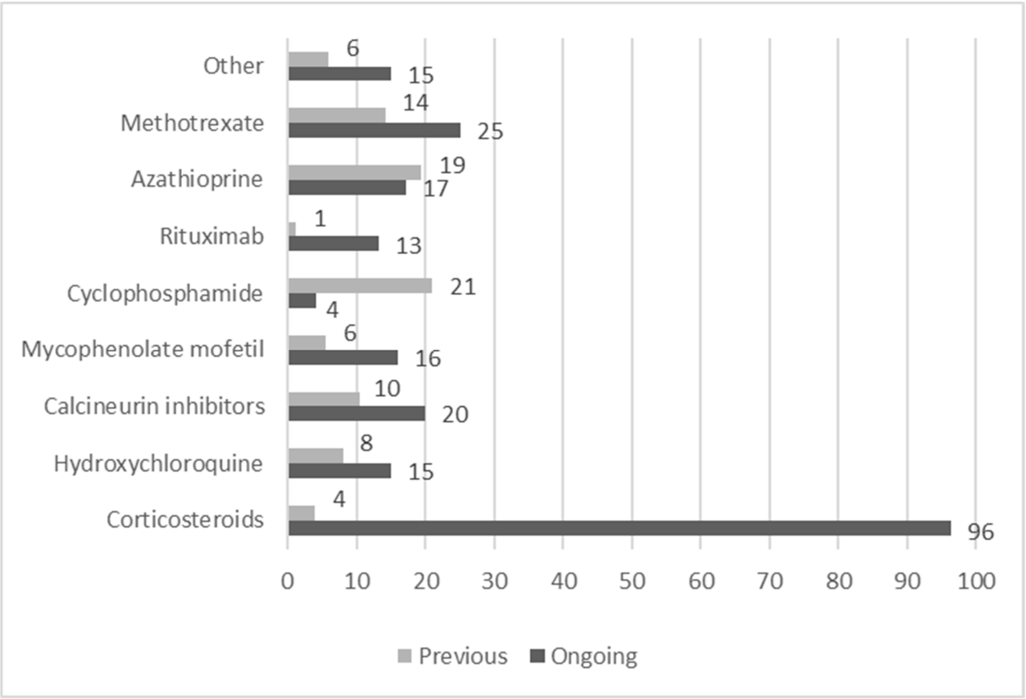


**Figure S2**: prevalence in percentage of ongoing and previous treatments of the included cohort of antisynthetase patients. Treatments have been included as a whole, by considering that our results confirmed the substantial homogeneinity of the syndrome.

**Table 1.** Cox proportional hazard model for estimating the time of ex-novo occurrence of triad findings.

| | **HR** | **95%CI** | **P** |
|---|---|---|---|
| **ARS** | | | |
| Anti-Jo-1 | 1 | | |
| Anti-PL-7 | 0.83 | 0.60-1.13 | 0.24 |
| Anti-PL-12 | **0.42** | **0.28-0.65** | **<0.001** |
| Anti-EJ ARS | 0.54 | 0.22-1.30 | 0.17 |
| Anti-OJ | 1.09 | 0.70-1.72 | 0.69 |
| **Sex (Male vs Female)** | 0.82 | 0.65-1.05 | 0.11 |
| **Age at onset (years)** | 1 | 0.99-1.01 | 0.26 |
| **Follow-up group (months)** | | | |
| 0-12 | 1 | | |
| 13-24 | **0.48** | **0.29-0.80** | **0.005** |
| 25-60 | **0.51** | **0.35-0.76** | **0.001** |
| >60 | **0.28** | **0.19-0.41** | **<0.001** |

hazard ratio (HR), 95% confidence interval (95% CI) and *p*-value (P) for each predictor are reported. Statistically significant differences ($p < 0.05$) in bold. Legend: ARS, antisynthetase antibodies
